# Parathyroidectomy in the Treatment of Childhood Hyperparathyroidism: A Single-Institution Experience

**DOI:** 10.3390/children13010064

**Published:** 2025-12-31

**Authors:** Seyithan Ozaydin, Serkan Sari, Emel Hatun Aytac Kaplan, Zumrut Kocabey Sutcu, Sevgi Yavuz, Hamit Yucel Barut, Huseyin Karatay, Burcu Esen Akkas

**Affiliations:** 1 Department of Pediatric Surgery, Liv Hospital Bahcesehir, Istinye University, Istanbul 34517, Turkey; 2 Department of General Surgery, Basaksehir Cam and City Hospital, University of Health Sciences, Istanbul 34480, Turkey; drserkansari@yahoo.com; 3 Department of Pediatric Endocrinology, Basaksehir Cam and City Hospital, University of Health Sciences, Istanbul 34480, Turkey; emel_ctf@hotmail.com (E.H.A.K.);; 4 Department of Pediatric Nephrology, Basaksehir Cam and City Hospital, University of Health Sciences, Istanbul 34480, Turkey; drsyavuz@gmail.com; 5 Department of Radiology, Basaksehir Cam and City Hospital, University of Health Sciences, Istanbul 34480, Turkey; 6 Department of Pathology, Basaksehir Cam and City Hospital, University of Health Sciences, Istanbul 34480, Turkey; huseyin140@gmail.com; 7 Department of Nuclear Medicine, Basaksehir Cam and City Hospital, University of Health Sciences, Istanbul 34480, Turkey; burcuesen@yahoo.com

**Keywords:** hyperparathyroidism, parathyroidectomy, primary hyperparathyroidism, renal hyperparathyroidism, pediatric surgery

## Abstract

**Highlights:**

**What are the main findings?**

- Hyperparathyroidism is rarely seen in childhood, and parathyroidectomy plays a crucial role in its treatment.
- There are very few studies in the literature that present the surgical outcomes of primary, secondary, and tertiary hyperparathyroidism cases together.

**What are the implications of the main findings?**

- Childhood parathyroidectomy is successfully performed in experienced institutions.
- Successful parathyroidectomy results are presented by comprehensively addressing all groups of childhood hyperparathyroidism.

**Abstract:**

**Purpose:** Hyperparathyroidism (HPT) is a condition marked by excessive secretion of parathyroid hormone (PTH), leading to disturbances in calcium, phosphate, and vitamin D metabolism. HPT is classified into primary (pHPT), secondary (sHPT), and tertiary (tHPT) types, which can cause systemic complications. Parathyroidectomy (PTX) remains the cornerstone treatment for pHPT and refractory cases of sHPT and tHPT. **Methods:** A retrospective review was conducted on 10 pediatric patients who underwent PTX for HPT at our clinic between 2016 and 2024. Demographic data, preoperative imaging, laboratory findings, surgical details, pathology reports, and postoperative outcomes were analyzed. Patients were categorized as having either pHPT (*n* = 6) or renal HPT (r-HPT; *n* = 4), which included one case of sHPT and three cases of tHPT. **Results:** The mean age of pHPT and r-HPT patients was 15 and 13 years, respectively. While 50% of pHPT patients were female, all r-HPT patients were female. Preoperative imaging localized parathyroid lesions using ultrasonography in all cases, but Sestamibi scintigraphy had a lower detection rate (66.7%). Minimally invasive parathyroidectomy was performed in single-gland pHPT cases, while bilateral neck exploration was used for multiglandular pHPT and all r-HPT cases. No intraoperative complications were observed. Postoperatively, all patients demonstrated normalized calcium, phosphate, and PTH levels with significant symptomatic improvement. Hungry bone syndrome developed in one r-HPT patient and was managed successfully. No recurrences were noted during an average follow-up of 39 months. **Conclusions:** PTX is a safe and effective treatment for pediatric HPT, providing excellent biochemical and clinical outcomes. Multidisciplinary collaboration is crucial in managing pediatric cases, particularly those with complex renal HPT.

## 1. Introduction

Hyperparathyroidism (HPT) is characterized by excessive secretion of parathyroid hormone (PTH) and is a cause-and-effect disorder of calcium (Ca), phosphorus (P), and vitamin D metabolism. According to the prevailing scientific consensus, the underlying mechanism of HPT is classified into three distinct types: primary hyperparathyroidism (pHPT), secondary hyperparathyroidism (sHPT), and tertiary hyperparathyroidism (tHPT). pHPT is predominantly caused by a solitary parathyroid adenoma, with rarer instances of multiple adenomas [1]. The sHPT results from excessive PTH secretion due to abnormal vitamin D and calcium metabolism, primarily caused by CRF but also by gastrointestinal malabsorption, liver disease, and pseudohypoparathyroidism. Persistent stimulation of the parathyroid glands in dialysis-dependent chronic renal failure (CRF) patients can lead to hyperplasia and, eventually, adenoma formation, termed tHPT [2,3].

Disturbances in the homeostasis of parathyroid hormone (PTH), calcium (Ca), phosphorus (P), and vitamin D result in a multitude of complications that affect diverse physiological systems, including the skeletal, renal, gastrointestinal, neuropsychiatric, soft tissue, and cardiovascular systems. Compared with adults, the clinical challenge in pediatric patients is more pronounced, as hypercalcemia may lead to significantly more severe neurodevelopmental consequences during critical periods of brain maturation.

The primary treatment for pHPT involves the surgical excision of the pathological parathyroid gland through parathyroidectomy (PTX) [4]. Conversely, sHPT and tHPT are generally managed through medical interventions, with surgery being reserved for cases that do not adequately respond to medical therapy [5].

Because the onset of symptoms is typically inscrutable in early childhood, patients frequently present to medical professionals at a late stage. A thorough evaluation and diagnosis by the relevant medical departments can result in a protracted process of obtaining a definitive evaluation [6].

The dearth of multidisciplinary teams comprising experienced surgeons across numerous medical centers poses a considerable challenge. The dearth of studies in the extant literature on rare childhood HPT cases, with the majority focusing on a single type, presents another challenge [7].

The objective of this study was to comprehensively evaluate the outcomes of parathyroidectomy (PTX) across all forms of hyperparathyroidism—primary (pHPT), secondary (sHPT), and tertiary (tHPT)—by analyzing surgical results from a single-center cohort and contextualizing these findings within the current literature.

## 2. Materials and Methods

All cases that were diagnosed, underwent surgical intervention, and were subject to postoperative follow-up were included in the present retrospective study. Conversely, cases with postoperative follow-up at external centers and those with incomplete file data were excluded from the study. Moreover, the Institutional Scientific Research Ethics Committee approved the study (Approval No. 2023.10.45, date 25 October 2023). The data collected encompassed a wide range of information, including demographic and clinical characteristics, preoperative localization studies, laboratory findings, surgical notes, postoperative follow-up data, and pathology reports.

In addition to cases of isolated single or multiple parathyroid adenomas (pHPT), cases of sHPT and tHPT occurring due to chronic renal failure (CRF) were evaluated under the title of renal hyperparathyroidism (r-HPT). For cases of pHPT involving a single gland, minimally invasive parathyroidectomy (MIP) was performed; subtotal parathyroidectomy with bilateral neck exploration (BNE) was employed for cases of pHPT involving multiple glands and for all r-HPT cases. The aforementioned approach entailed the excision of three and a half pathological glands.

Initial evaluation of all cases was conducted using ultrasonography. The standard diagnostic approach included the use of intraoperative intact parathyroid hormone assay biopsy (iiPTH/washout) procedures to achieve differential and definitive diagnosis of the lesion. In light of the potential risks associated with parathyroidomatosis, the procedure was performed with particular caution and scheduled within 1–2 days prior to surgery. TC-99m sestamibi scintigraphy was utilized for two primary purposes: localization of the parathyroid lesion and identification of possible ectopic glandular tissue.

A comprehensive data set was systematically collected, encompassing preoperative and postoperative serum calcium (Ca), phosphorus (P), and parathyroid hormone (PTH) levels; preoperative ultrasonography findings; Tc-99m sestamibi scintigraphy/single-photon emission computed tomography (S-mibi/SPECT-CT) reports; washout/fine-needle aspiration biopsy (W/FNA) results; preoperative preparation protocols; intraoperative additional interventions when required; perioperative complications; and postoperative follow-up outcomes. A meticulous review of the pathology reports was conducted. Subsequent analyses of clinical and biochemical outcomes were based on postoperative follow-up evaluations at 6 and 12 months.

### Statistical Analysis

The limited sample size and lack of data uniformity precluded a comprehensive comparative statistical analysis. Instead, descriptive statistics were used to summarize data, with continuous variables presented as means, standard deviations, medians, minimums, and maximums. Categorical data were expressed as frequencies and percentages. Analyses were conducted using MedCalc^®^ Statistical Software version 22.009 (MedCalc Software Ltd., Ostend, Belgium; https://www.medcalc.org; accessed on 20 October 2023).

## 3. Results

The study included 10 patients: 6 (60%) had pHPT, 1 (10%) had sHPT, and 3 (30%) had tHPT. The latter 4 cases (40%) were categorized as r-HPT. The mean age of patients diagnosed with pHPT was 15 years, while the mean age of patients diagnosed with r-HPT was 13 years. Among the patients with pHPT, 50% were female, whereas all r-HPT cases involved female patients. Patients with pHPT most commonly presented with gastrointestinal, neuropsychiatric, and musculoskeletal complaints, whereas r-HPT cases primarily exhibited symptoms associated with renal osteodystrophy, including issues related to the urinary and musculoskeletal systems.

Supplementary Table S1 presents the demographic data, clinical features, and diagnostic and therapeutic management for the 10 cases in our pediatric HPT/PTX series. Table 1 summarizes the demographics and system-related symptoms of the pHPT and r-HPT groups. A meticulous review of the medical histories of the six patients in the pHPT group revealed no significant findings. However, in the r-HPT group, one patient exhibited bilateral dysplastic multicystic kidneys, while another had a medical history marked by apert hydrocephalus, ventriculoperitoneal shunt placement, meningomyelocele repair, spina bifida, and associated neurogenic bladder, as well as bilateral vesicoureteral reflux. The etiology of the two remaining cases of r-HPT with CRF remained unclear.

With respect to family history, one patient with pHPT had a father with urinary stone disease, and another had a sibling with growth hormone deficiency. In the r-HPT group, only one patient had a sibling with CRF. Genetic screening revealed no molecular or genetic abnormalities in any of the patients.

The mean duration of symptoms was significantly longer in r-HPT cases (96 months) than in pHPT cases (10.8 months). Preoperative laboratory findings revealed that, in the pHPT patients, the mean calcium (Ca), phosphorus (P), and parathyroid hormone (PTH) levels were 12.9 ± 1 mg/dL, 2.5 ± 0.4 mg/dL, and 259.5 ± 276.7 pg/mL, respectively. For patients undergoing R-HPT, these levels were 9.2 ± 0.9 mg/dL, 6.2 ± 1.5 mg/dL, and 3861.5 ± 1336.7 pg/mL, respectively.

Regarding imaging studies, preoperative US identified parathyroid adenomas in all cases of pHPT. In contrast, hyperplastic/adenomatous findings were observed in all r-HPT cases except one. W/FNAB was performed in all pHPT cases, confirming PTH levels exceeding 5000 pg/mL. Preoperative S-mibi/S-CT revealed no uptake in two pHPT cases (33.3%) and one r-HPT case (25%).

The surgical approaches employed were contingent upon the clinical presentation. MIP was performed in five cases of pHPT with single-gland involvement, while BNE was conducted in one pHPT case with multi-gland involvement and in all four r-HPT cases. An intubation tube-compatible neuromonitor (TechnoCath, Yalova, Turkey) (Dr. Langer Avalanche, serial number: 789.09.2015, Germany) was utilized in all cases. Intraoperative PTH monitoring (io-PTH) was performed in only one pHPT case due to two-gland involvement. Furthermore, frozen-section biopsies were performed in one pHPT and one r-HPT case to confirm tissue pathology. Parathyroid adenoma was excised in all cases of pHPT, while partial thymectomy was additionally performed in two cases of lower lobe origin.

All r-HPT cases underwent four-gland exploration and three-and-a-half-gland excision. Moreover, one underwent unilateral thyroidectomy, and three also underwent unilateral (partial/subtotal) thymectomy. The lower lobe was preferred for the half gland left, as it is generally safer for the laryngeal nerve in a possible second exploration. However, in two cases in which the lower lobe glands were markedly hyperplastic, the upper lobe, which had a somewhat more normal appearance, was chosen. After excision, the remaining gland was marked with a metal clip. The three cases of pHPT originated in the left lower lobe, whereas one originated in the right lower lobe, one in the right upper lobe, and one in both the right lower and upper lobes.

In all but 2 cases of pHPT, a minivac drain was placed in the lounge and, on average, withdrawn on postoperative day 1. No intraoperative complications or postoperative complications, such as bleeding, nerve injury, hematoma, or infection, were encountered. Postoperatively, one tHPT case developed hungry bone syndrome (HBS), which was successfully treated with medical intervention. Another tHPT case experienced transient hyperthyroidism, likely associated with the use of povidone-iodine antiseptic; this condition was resolved with appropriate medical management.

In the early postoperative period, the mean Ca value was 8.9 ± 0.2 mg/dL, the mean *p* value was 2.5 ± 0.4 mg/dL, and the mean PTH value was 36.5 ± 12 pg/mL in patients with pHPT. Conversely, in r-HPT cases, the mean Ca value was 8.3 ± 0.5 mg/dL, the mean *p* value was 4.3 ± 1.3 mg/dL, and the PTH value was 57 ± 44.9 pg/mL.

**Figure 1:**
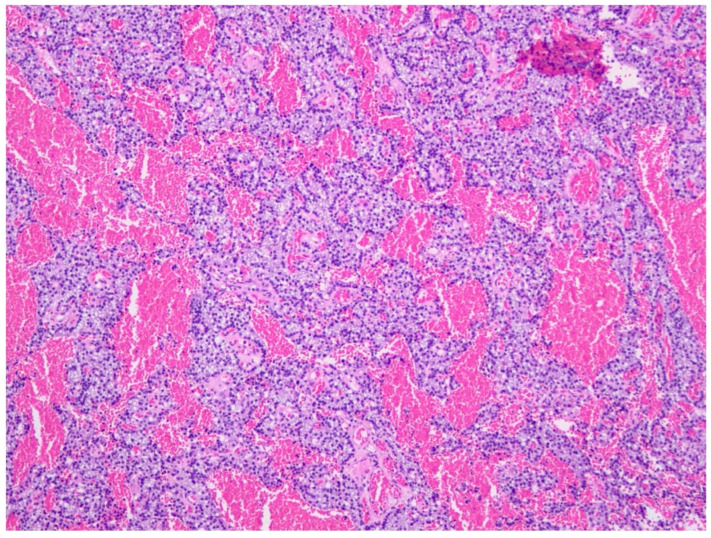
Histopathological image of parathyroid adenoma seen in primary HPT.

**Figure 2:**
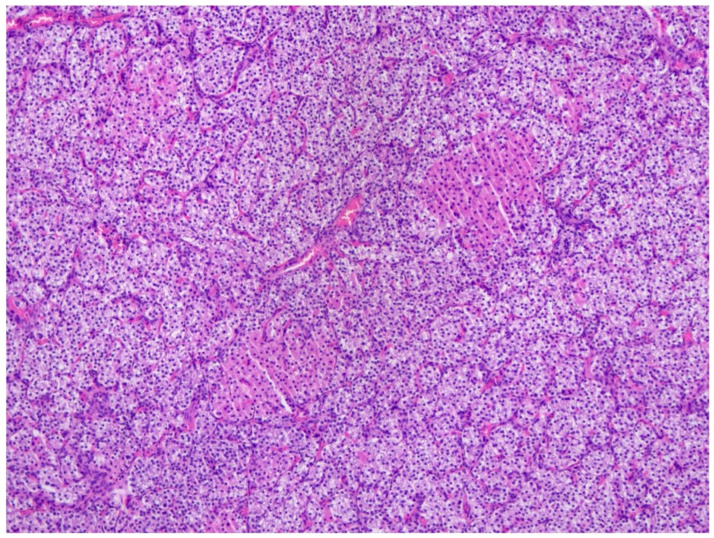
Histopathological image of parathyroid hyperplasia seen in secondary HPT.

A common difficulty encountered in histopathological examination of specimens is distinguishing adenoma from hyperplasia. The evaluation of these findings is conducted in conjunction with clinical and laboratory findings. Furthermore, while adenoma growth is characterized by a nodular pattern rather than diffuse expansion, cellular proliferation is observed in hyperplasia. In the context of this approach, a subsequent examination of the pathology reports revealed that the lesions were evaluated as parathyroid adenomas in all cases of pHPT (*n* = 6) (Figure 1). In r-HPT cases, all removed glands were hyperplastic, particularly in sPHT cases (Figure 2). In three cases of tHPT, all glands were found to be hypercellular adenomatous nodules. Histopathological images of the preparations in Figure 1 and Figure 2 were obtained with limited resolution due to technical difficulties. The weight of the pathology specimen in pHPT cases was found to be mean ± SD: 480 ± 147.5 mg (*n* = 7), while in r-HPT cases, it was mean ± SD: 453.1 ± 549.4 mg (*n* = 16). The volume (mm^3^) in pHPT cases was mean ± SD: 417.5 + 234.9 (*n* = 8); in r-HPT cases, whereas it was mean ± SD: 512.1 + 464.3 (*n* = 16).

The average hospital stay was 2 ± 0 days for pHPT patients and 11 ± 2.7 days for r-HPT patients. The mean follow-up period after PTX was 50 ± 32.6 months in pHPT cases and 22.5 ± 3 months in r-HPT cases. In all cases, no problems requiring reoperation were encountered. At the sixth postoperative month, the mean Ca value in pHPT cases was 9.2 ± 0.5 mg/dL, the mean *p* value was 3.6 ± 0.6 mg/dL, and the mean PTH value was 49.5 ± 8. 7 pg/mL, while the mean values of r-HPT cases were 8.8 ± 0.7 mg/dL for Ca, 4.3 ± 1.3 mg/dL for P, and 38 ± 28.9 pg/mL for PTH. At the 12th postoperative month, the mean Ca value of pHPT cases was 8.7 ± 0.2 mg/dL, the *p* value was 3.5 ± 0.2 mg/dL, and the PTH value was 44.8 ± 5.5 pg/mL, while in r-HPT cases, the mean values of Ca was 9 ± 0.9 mg/dL, P 4.8 ± 1.1 mg/dL, and PTH 30.5 ± 23.8 pg/mL (Table 2).

**Figure 3:**
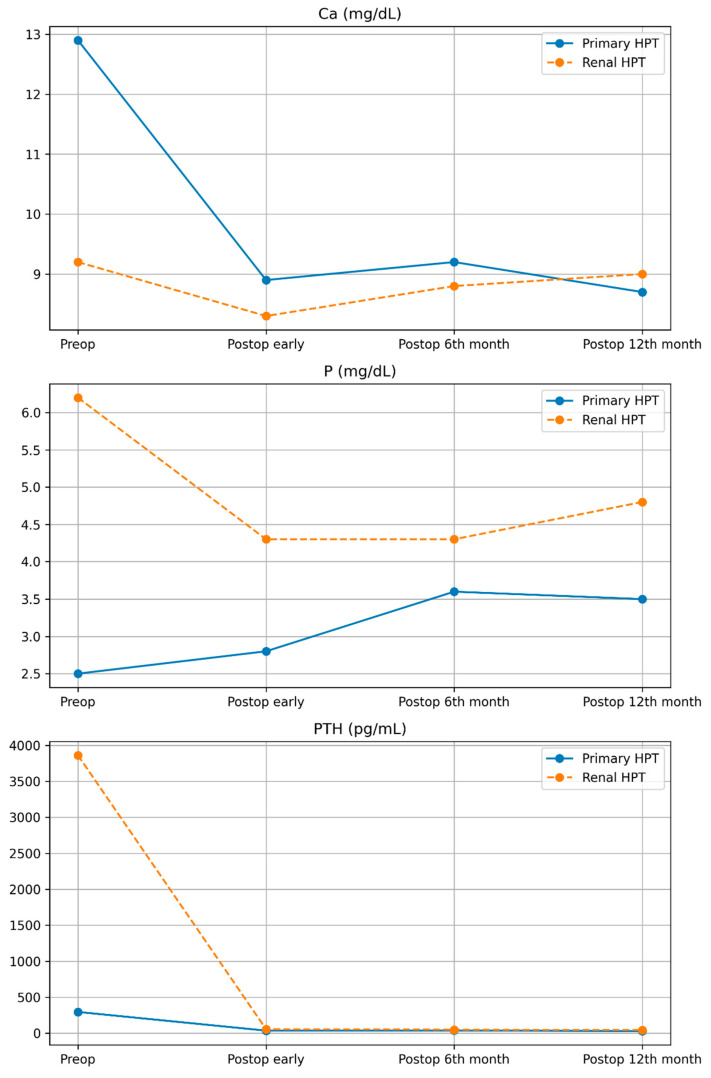
Trends of blood Ca, P, and PTH levels in the patients with primary and renal hyperparathyroidism.

The preoperative and postoperative trends of Ca, P, and PTH levels are displayed in Figure 3. At the final follow-up visits, all patients reported no complaints, as in the preoperative period, and a significant improvement in their overall well-being. Furthermore, no new bone fracture findings were observed, particularly in r-HPT cases.

## 4. Discussion

This single-center study evaluated surgical outcomes in 10 pediatric patients with hyperparathyroidism, including 6 with primary and 4 with renal forms. Time to diagnosis was shorter in primary cases, while parathyroid hormone and phosphorus levels were higher in renal hyperparathyroidism. Ultrasonography successfully localized all lesions, whereas S-mibi/S-CT showed limited uptake in some patients. Minimally invasive parathyroidectomy was preferred for most primary cases, while bilateral neck exploration was performed for multiglandular disease and all renal cases. Apart from one instance of postoperative hungry bone syndrome, no significant complications were observed, and long-term follow-up demonstrated biochemical normalization and clinical improvement.

### 4.1. Demographics, Etiology, Clinical Presentation, and Diagnosis

pHPT is rare in pediatric populations, with an incidence of 0.5–5 per 100,000, compared to 20–100 per 100,000 in adults [8,9,10,11]. Most reports indicate a female predominance, likely due to hormonal factors influencing parathyroid growth and function [8,12]. In contrast to previous reports, our cohort demonstrated an equal male-to-female ratio. This apparent gender parity in pHPT cases may not accurately represent the actual population distribution, as our institution serves as a referral center and receives cases from multiple external hospitals. Additionally, this finding may be influenced by the relatively small sample size, potential regional genetic factors, and sex-based differences in healthcare-seeking behaviors among pediatric patients.

The etiology of pHPT has been extensively discussed in the literature. It has been reported that 68–81.5% of cases are sporadic, while 8.5–12.8% are familial, and 5.8–11.5% are associated with genetic or molecular abnormalities. These abnormalities are often linked to syndromes such as Multiple Endocrine Neoplasia (MEN-1, MEN-2A, MEN-4) or mutations in genes such as CASR, CDKN1A, CDKN1B, CDKN2C, and RET [13,14,15]. Consistent with prior studies emphasizing the sporadic nature of pediatric pHPT, this study found no familial or syndromic cases, and genetic screening did not reveal any molecular abnormalities. This further underscores that genetic testing remains valuable in pediatric pHPT workups, but its diagnostic yield may be low in sporadic cases.

The literature indicates that about 80% of cases of pHPT involve a single gland [16]. Multiglandular involvement is more commonly associated with syndromic or familial cases [13,15]. In our cohort, the majority of cases involved a single gland, as reported in the literature.

In pHPT, the clinical presentation is primarily symptomatic, with 64–96% of cases demonstrating significant clinical findings. These symptoms commonly involve the musculoskeletal, gastrointestinal, neuropsychiatric, and urinary systems. Patients often present with muscle cramps, difficulty walking, bone deformities, bone fractures, nausea, vomiting, abdominal pain, gastroenteritis, headaches, fatigue, depression, or nephrolithiasis [4,8,12,17,18]. In agreement with previous studies, all pHPT cases in this study were symptomatic, with musculoskeletal, neuropsychiatric, and gastrointestinal complaints being the most frequent. Notably, nephrolithiasis and nephrocalcinosis, reported in about 40% of pediatric cases, were absent in this cohort, possibly due to earlier diagnosis or differences in genetic predisposition [19].

Regarding r-HPT, CRF prevalence in adults is estimated at 40–70 per million, but it is much rarer in children (0.5–5 per million). Nevertheless, a significant proportion of pediatric CRF patients develop r-HPT (sHPT or tHPT), which, if untreated, can cause severe bone deformities and growth retardation [20]. Our study confirms this trend, with pediatric r-HPT patients presenting with bone deformities and leg pain as major clinical concerns. In particular, the postoperative decrease in parathyroid hormone (PTH) levels prevented bone deformities from occurring in the cases under study. Furthermore, patients reported that the leg pain they felt before surgery was no longer present.

### 4.2. Biochemical Profiles in Pediatric HPT

The biochemical profile in pediatric pHPT typically includes Ca levels of 11.5–12.1 mg/dL, P levels of 2.5–3.4 mg/dL, and PTH levels of 91.8–177.5 pg/mL [4,8,12,17]. This study’s findings were broadly consistent with these values, though PTH levels were notably higher. This elevation may be due to more advanced disease at diagnosis or variability in PTH assay sensitivity across institutions.

Studies report preoperative serum levels in r-HPT as Ca 9.6 ± 1.7 mg/dL, P 3.9 ± 1.1 mg/dL, and PTH 1240.1 ± 160.1 pg/mL [5]. In our r-HPT cases, PTH levels were significantly higher, and elevated phosphorus levels indicated severe disease.

### 4.3. Preoperative Imaging and Intraoperative Localization/Monitoring

Accurate localization of parathyroid adenomas is critical for surgical planning in cases with pHPT. The clinical findings and laboratory values are sufficient for establishing a diagnosis of HPT. However, to protect against the potential serious complications of surgical procedures, in addition to routine ultrasound, sestamibi is extremely important for determining the location of the lesion and the presence of ectopic tissue, if any. Preoperative imaging using a combination of US and S-mibi/S-CT has been reported to have a sensitivity of 82–91.4% in localizing parathyroid lesions [8,12,17,21]. The clinical findings and laboratory values are sufficient for establishing a diagnosis of HPT. However, to protect against the potential serious complications of surgical procedures, in addition to routine ultrasound, sestamibi is extremely important for determining the location of the lesion and the presence of ectopic tissue, if any.

On the other hand, factors such as technical characteristics and experience can influence the accuracy of results regarding the lesion’s presence, size, and location. The sensitivity is exceptionally high in single-gland involvement, with detection rates ranging from 89% to 94%, but it decreases in cases involving multiple glands or small lesions [22]. In one study, the US alone achieved 100% positive localization in its cohort. However, concordance with S-mibi was observed in only 93.8% of cases, and the authors concluded that S-mibi mainly improves specificity in suspected multiglandular disease and should be carefully evaluated in pediatric cases to minimize radiation exposure [23]. Multiglandular involvement in pHPT is reported in 7–33% of cases, with varying rates depending on imaging methods. Multiglandular disease rates were 5.7%, 11%, and 21% for positive findings on both, one, or neither imaging modality, respectively [24]. Careful attention is needed when operating on small or multiple affected glands. Surgical outcomes were comparable, even in imaging-negative cases. The US successfully localized all parathyroid lesions in this study, reinforcing its reliability. At the same time, S-mibi/S-CT failed to detect uptake in 33.3% of pHPT cases, supporting prior reports that the necessity of S-mibi/S-CT in single-gland involvement remains debatable. Li et al. reported that, despite advances in diagnostic tools, the average time to diagnosis of pHPT cases remains 41 months [25]. In contrast, our cohort’s mean duration from symptom onset to diagnosis was 10.8 months. Preoperative imaging is also essential in r-PHT to identify ectopic glands and minimize unnecessary dissections [26,27].

Preoperative imaging effectively guided surgical planning in all of our cases except for one with multiglandular involvement. Despite advances, 10–20% of pHPT lesions were reported to remain unlocalized preoperatively, similar to our study, leading to intraoperative techniques like gamma-probe-guided localization and io-PTH [17,26,28,29]. Regarding io-PTH, both the American Association of Endocrine Surgeons (AAES) and the European Society of Endocrine Surgeons (ESES) endorse io-PTH, particularly in discordant imaging cases or reoperative PTX [28]. However, its routine use remains debated due to cost and logistical challenges. While intraoperative PTH testing can be conducted rapidly using immunochemical devices installed in the operating theater, this approach is more expensive than central laboratory testing. The major drawback of central laboratory PTH testing is the delay in obtaining results, typically requiring 25–30 min [21]. In the case of multiglandular involvement, io-PTH and frozen-section biopsy were performed to guide surgical decisions. Our approach reflects this controversy: we opted against routine io-PTH use in all cases, prioritizing its use only when necessary. Gamma-probe-guided localization techniques have proven safe and effective in reoperative PTX and sHPT [29,30]. Aygün et al. noted no consensus regarding their routine use [21]. Consistent with this, gamma probes were not routinely employed in our cohort.

### 4.4. Management, Complications, and Outcomes of Ptx in Pediatric Hpt

A multidisciplinary approach is essential in managing HPT, particularly in pediatric cases where surgical experience may be limited. Collaboration among endocrinology, radiology, nuclear medicine, and pathology departments is critical, and surgeries should be performed under the supervision of an experienced endocrine surgeon [4]. Our series evaluated all cases collaboratively with pediatric endocrinology, radiology, nuclear medicine, and pathology teams. Surgeries were performed under the supervision of an experienced endocrine surgeon. Although the small sample size is a limitation of our series, the outcomes align well with those reported in the literature.

Intraoperative neuromonitoring (NIM 3.0, Medtronic, Minneapolis, MN, USA) is widely used in PTX procedures to prevent nerve injury. Despite transient vocal cord paralysis in two cases in a previous study, no permanent paralysis was observed [26]. Similarly, intraoperative neuromonitoring was employed in all our surgeries, and no cases of transient or permanent laryngeal nerve injury were recorded.

PTX remains the gold standard for treating pHPT. Traditionally, BNE involved a large incision to explore all parathyroid glands. However, advancements in preoperative imaging, intraoperative gamma-probe technology, and io-PTH have facilitated a shift toward MIP for single-gland disease, allowing for a more targeted approach with a smaller incision [26]. MIP is particularly effective for solitary parathyroid adenomas, though multiglandular disease or ectopic gland locations—present in approximately 12% of cases—may necessitate a broader surgical field [17]. Studies report MIP usage rates ranging from 40% to 78%, with io-PTH playing a critical role, especially when ectopic glands are suspected [4,12]. In our cohort, the predominance of single-gland disease allowed MIP in most cases, whereas multiglandular involvement required BNE, consistent with the literature. Walsh et al. identified essential factors contributing to MIP success, including high-resolution US, S-mibi/S-CT, and io-PTH monitoring. More than a 50% reduction in PTH levels at 5–30 min post-excision or normalization to <65 pg/mL is considered adequate for confirming surgical success [31]. Our findings align with these criteria, as io-PTH monitoring was selectively applied in a multiglandular PTH case, aiding surgical decision-making.

A systematic review comparing MIP and BNE reported similar cure rates (97% vs. 98%) but found MIP to have a superior safety profile, with lower rates of hypocalcemia (2.3% vs. 14%) and laryngeal nerve injury (0.3% vs. 0.9%) [1]. Another study confirmed these benefits, highlighting MIP’s association with shorter operative time (64 vs. 103 min), reduced pain, lower analgesic requirements, and improved cosmetic outcomes [21]. A 2021 systematic review emphasized MIP’s increasing adoption due to its high success rates and reduced complications, concluding that MIP offers a success rate comparable to BNE but provides additional advantages, including fewer postoperative complications, shorter hospital stays, and superior cosmetic outcomes. The review also reported that both the American Association of Endocrine Surgeons (AAES) and the European Society of Endocrine Surgeons (ESES) guidelines for io-PTH recommend its use, especially in cases where preoperative localization by US-mibi/S-CT is not compatible or re-operative parathyroidectomy (PTX) is required; the true benefit of io-PTH monitoring remains a subject of debate among endocrine surgeons [28]. However, BNE remains the gold standard for multiglandular disease, MEN-1, or cases with negative preoperative imaging [21]. Our findings support this distinction: single-gland disease cases underwent MIP, while BNE was reserved for multiglandular involvement to ensure optimal surgical outcomes.

Histopathological findings in pHPT generally confirm parathyroid adenomas, with some cases of hyperplasia. Lesion weights reported in the literature range from 130 to 2440 mg [8,17]. Our findings align with these reports: all excised lesions were confirmed as adenomas, with an average lesion weight of 480 mg.

Although outpatient PTX has been deemed safe for pediatric pHPT patients [32], we opted for a more cautious approach. All patients in our study were hospitalized for an average of two days, consistent with the need for careful postoperative monitoring. Following PTX, studies report postoperative improvements in insomnia, dyspepsia, quality of life, neuropsychiatric symptoms, cardiovascular health, reduced nephrolithiasis and fracture risks, and increased bone mineral density [21,33]. Similarly, our patients reported complete resolution of symptoms and improved overall well-being postoperatively.

For r-HPT, first-line treatment involves vitamin D and cinacalcet therapy [34,35]. PTX is considered in 5–25% of cases unresponsive to medical management, as uncontrolled r-HPT can cause severe osteodystrophy and vascular calcifications [5,36,37]. Indications for PTX in sHPT include severe hypercalcemia, progressive bone disease, and pruritus, while in tHPT, persistent hypercalcemia and kidney dysfunction post-transplant warrant surgical intervention [20]. A study by Seong Hoon Kim et al. outlined PTX indications in sHPT as medical therapy resistance, symptomatic disease, and persistently elevated PTH, whereas in tHPT, indications include persistent hypercalcemia, nephrolithiasis, and declining kidney graft function [26]. Our findings align, as all r-HPT patients in our cohort met surgical indications, including persistently elevated PTH and clinical symptoms.

Surgical options for r-HPT include PTX with autotransplantation or subtotal PTX (removal of 3.5 glands). Total PTX without autotransplantation is not recommended in children due to high morbidity risks. Seong Hoon Kim et al. preferred a subtotal PTX approach [26]. In our r-HPT cases, four glands were identified, and subtotal PTX with 3.5 glands excised was performed. Autotransplantation was not employed in any case. Unlike reports advocating autotransplantation in subtotal PTX, our findings indicate that avoiding it did not result in postoperative complications or additional surgical interventions. Elijah Kakani et al. highlighted the critical role of PTX in managing inadequately controlled r-HPT cases, but noted that evidence supporting its effect on mortality reduction is mainly derived from retrospective and observational data. They cautioned that PTX carries potential risks, including persistent or recurrent HPT and hypoparathyroidism, which necessitate careful long-term monitoring.

Furthermore, they raised concerns that transitioning from a high-turnover to a low-turnover bone state post-PTX may predispose patients to vascular calcifications, complicating calcium homeostasis in CRF patients with mineral bone disease [38]. Our results support the efficacy of subtotal PTX in reducing PTH levels without leading to overt hypoparathyroidism, reinforcing the importance of preserving well-vascularized parathyroid tissue to mitigate post-PTX complications. None of our patients experienced new bone fractures, and significant clinical improvements in leg pain and mobility were observed.

No significant complications occurred in our pHPT cohort. Long-term follow-up showed biochemical normalization and symptom improvement. On the other hand, one patient with tHPT developed postoperative HBS, a known complication due to high post-PTX osteoblastic activity. This condition was successfully managed with appropriate medical interventions, and the patient recovered without complications. Our results support prior findings that elevated PTH levels are a risk factor for HBS, emphasizing the importance of preoperative optimization. Curtis Hanba et al. reported that male patients, younger children, and those with pre-existing kidney disease had more prolonged and complex postoperative courses, including mental status changes, infections, and respiratory complications [39]. In contrast, all of our r-HPT patients were female, yet they still experienced extended hospital stays, primarily due to the need for fluid and electrolyte management during dialysis rather than postoperative complications.

A large-scale study comparing PTX outcomes in pHPT and r-HPT patients found no significant age differences between the groups, although a female predominance was observed in pHPT. It reported that r-HPT patients had longer operative times, higher estimated blood loss, and longer hospital stays than pHPT patients. The study also found that in pHPT, lesions were most frequently located in the right lower and left lower glands, whereas in r-HPT, the preserved gland was typically in the left upper position. Preoperative PTH levels were significantly higher in r-HPT patients (1242.1 ± 1075 pg/mL) than in pHPT patients (161.6 ± 95.4 pg/mL). Postoperative PTH levels at 6 and 12 months were comparable between the two groups. Preoperative calcium levels were higher in pHPT cases (10.9 ± 0.9 mg/dL vs. 9.6 ± 1.7 mg/dL), while postoperative calcium levels normalized immediately in pHPT patients but took longer in r-HPT patients, with hypocalcemia persisting for up to six months postoperatively [24]. Consistent with these findings, our study also observed significantly higher preoperative PTH levels in r-HPT patients compared to pHPT patients. However, unlike the reported delayed calcium normalization in r-HPT cases, our cohort maintained stable postoperative calcium levels within normal ranges at the 6- and 12-month follow-ups. The follow-up periods for our r-HPT cases are shorter compared to our pHPT cases (22.5 ± 3 months compared to 50 ± 32.6 months). This is due to the fact that r-HPT cases have been referred to our center mostly in recent years.

## 5. Limitations of the Study

The retrospective single-center design, the relatively small sample size, the absence of statistical comparisons, and the lack of comprehensive genetic testing in some patients constitute important limitations that may affect the generalizability of our findings. Consequently, the execution of additional multicenter studies comprising larger cohorts is imperative to validate and expand upon these findings.

## 6. Conclusions

Pediatric HPT poses notable diagnostic and therapeutic complexities, compounded by its rarity and the broad spectrum of clinical presentations. In this series, pHPT occurred equally in males and females—contrary to the predominantly female trend reported elsewhere—while r-HPT affected only females. Despite these differences, all children in both groups presented with significant biochemical derangements, including markedly elevated PTH levels, underscoring the urgency of early recognition and intervention. Surgical management tailored to disease type and gland involvement proved highly effective: MIP was optimal for single-gland pHPT, while BNE with subtotal parathyroidectomy was preferred for multiglandular disease and r-HPT. Meticulous preoperative imaging—particularly ultrasound, which localized all lesions—minimized the need for additional imaging methods in straightforward single-gland pHPT. Complications were minimal, with only one postoperative HBS in an r-HPT patient, which was successfully managed medically. No recurrences were observed during long-term follow-up, signaling robust and durable outcomes.

These findings reinforce the efficacy of targeted surgical approaches and highlight the pivotal roles of multidisciplinary collaboration and vigilant postoperative monitoring. Refining diagnostic algorithms, optimizing intraoperative techniques, and honing perioperative care will be essential to improve further outcomes and quality of life for pediatric HPT patients.

## Figures and Tables

**Table 1:** Demographics and Presenting Symptoms.

	pHPT (*n* = 6)	r-HPT (*n* = 4)
Age (years) (Mean ± SD)	15 ± 3.1	13 ± 4.4
Gender, *n* (%)		
Male	3 (50%)	0 (0%)
Female	3 (50%)	4 (100%)
Symptom Duration (months) (Mean ± SD)	10.8 ± 7.5	96 ± 46
System-related Symptoms		
Neuropsychiatric		
Headache	3	0
Dizziness	1	0
Irritability	2	0
Forgetfulness	1	0
Fatigue	1	0
Seizures	0	1
Gastrointestinal		
Abdominal pain	4	0
Nausea/Vomiting	1	0
Gastroenteritis	1	0
Weight gain	1	0
Musculoskeletal		
Leg pain/Burning	4	1
Cramping/Spasms	2	0
Walking difficulty	0	2
Bone deformities (X-bone)	0	2
Fractures	0	2
Urinary		
Nephrolithiasis	1	0
Chronic kidney failure	0	4

**Table 2:** Laboratory Features.

Variable (Mean ± SD)	pHPT (*n* = 6)	r-HPT (*n* = 4)
Calcium (Ca, mg/dL)		
Preoperative	12.9 ± 1	9.2 ± 0.9
Postoperative (Early)	8.9 ± 0.2	8.3 ± 0.5
Postoperative (6 months)	9.2 ± 0.5	8.8 ± 0.7
Postoperative (12 months)	8.7 ± 0.2	9 ± 0.9
Phosphorus (P, mg/dL)		
Preoperative	2.5 ± 0.4	6.2 ± 1.5
Postoperative (Early)	2.8 ± 0.6	4.3 ± 1.3
Postoperative (6 months)	3.6 ± 0.6	4.3 ± 1.3
Postoperative (12 months)	3.5 ± 0.2	4.8 ± 1.1
PTH (pg/mL)		
Preoperative	259.5 ± 276.7	3861.5 ± 1336.7
Postoperative (Early)	36.5 ± 12.1	57 ± 44.9
Postoperative (6 months)	49.5 ± 8.7	38 ± 28.9
Postoperative (12 months)	44.8 ± 5.5	30.5 ± 23.8
Specimen Weight (mg), n	480 ± 147.57	453.1 ± 549.416
Specimen Volume (mm^3^)	417.5 ± 234.9	512.1 ± 464.3
Hospital Stay (days)	2 ± 0	11 ± 2.7
Follow-up Period (months)	50 ± 32.6	22.5 ± 3

## Data Availability

The data that support the findings of this study are available from the corresponding author upon reasonable request.

## Supplementary Materials

The following supporting information can be downloaded at: https://www.mdpi.com/article/10.3390/children13010064/s1, Table S1: Case Details.

## Abbreviations

The following abbreviations are used in this manuscript:

BNE	Bilateral neck exploration
CRF	Chronic renal failure
HBS	Hungry bone syndrome
HPT	Hyperparathyroidism
Io-PTH	Intraoperative parathyroid hormone monitoring
MIP	Minimally invasive parathyroidectomy
pHPT	Primary hyperparathyroidism
PTH	Parathyroid hormone
PTX	Parathyroidectomy
r-HPT	Renal hyperparathyroidism
S-CT	Single-photon emission computed tomography
sHPT	Secondary hyperparathyroidism
S-mibi	Tc 99 sestamibi scintigraphy
tHPT	Tertiary hyperparathyroidism
US	Ultrasonography
W/FNA	Washout/Fine needle aspiration biopsy
