# Supplementary material for: Parathyroidectomy in the Treatment of Childhood Hyperparathyroidism: A Single-Institution Experience
Source: Children (Basel). 2025 Dec 31;13(1):64. doi: 10.3390/children13010064 (PMC12840163; doi:10.3390/children13010064)
Supplement: Supplementary file 1 [file children-13-00064-s001.zip › children-4040559-supplementary.pdf]

Supplementary Table S1: Case Details

Case	1	2	3	4	5	6	7	8	9	10
**HPT Type**	pHPT	pHPT	pHPT	pHPT	pHPT	pHPT	sHPT	tHPT	tHPT	tHPT
**Age (years)**	17	14	16	9	17	17	15	8	11	18
**Sex**	Male	Female	Female	Female	Male	Male	Female	Female	Female	Female
**Symptom Duration (months)**	24	2	6	12	9	12	36	84	132	132
**Medical History**	None	None	None	None	None	None	Bilateral multicystic CKD	Hydrocephalus surgery, VP shunt, meningomyelocele, bilateral VUR, CKD	CKD	CKD
**Family History**	None	None	Father: kidney stones	Brother: growth hormone deficiency	None	None	Sibling: CKD	None	None	None
**Genetic/Molecular Abnormality**	None detected	None detected	None detected	None detected	None detected	None detected	None detected	None detected	None detected	None detected
**Preoperative Ca (mg/dL)**	13.4	11.5	13.4	13.9	11.9	13.5	10.4	8.3	9.3	8.8
**Preoperative P (mg/dL)**	2.4	2.3	1.8	2.9	2.9	2.6	7.7	5.2	7.3	4.7
**Preoperative PTH (pg/mL)**	184	144	821	127	104	177	3019	5000	5000	2427
**Ultrasound (mm)**	Right 12×7×5	Left 10×8×2	Right lower 15×12×25	Left lower 6×6×4	Left lower 6.7×4.6×3	Right lower 9×6×4	Normal	4 glands: Right 30×6×1, right lower 6×3×2, left lower 7×4×3 and 7×5×4	Right lower 11×6.5×5, left lower 7.5×4×3	Right lower 20×10×10, left lower 13×5×5
